# Strategies and molecular tools to fight antimicrobial resistance: resistome, transcriptome, and antimicrobial peptides

**DOI:** 10.3389/fmicb.2013.00412

**Published:** 2013-12-31

**Authors:** Letícia S. Tavares, Carolina S. F. Silva, Vinicius C. de Souza, Vânia L. da Silva, Cláudio G. Diniz, Marcelo O. Santos

**Affiliations:** ^1^Department of Biology, University of Juiz de ForaJuiz de Fora, Brazil; ^2^Department of Microbiology, Immunology and Infectious Diseases, University of Juiz de ForaJuiz de Fora, Brazil

**Keywords:** resistome, transcription, genetic, molecular modeling, antimicrobial peptides, NGS applications

## Abstract

The increasing number of antibiotic resistant bacteria motivates prospective research toward discovery of new antimicrobial active substances. There are, however, controversies concerning the cost-effectiveness of such research with regards to the description of new substances with novel cellular interactions, or description of new uses of existing substances to overcome resistance. Although examination of bacteria isolated from remote locations with limited exposure to humans has revealed an absence of antibiotic resistance genes, it is accepted that these genes were both abundant and diverse in ancient living organisms, as detected in DNA recovered from Pleistocene deposits (30,000 years ago). Indeed, even before the first clinical use of antibiotics more than 60 years ago, resistant organisms had been isolated. Bacteria can exhibit different strategies for resistance against antibiotics. New genetic information may lead to the modification of protein structure affecting the antibiotic carriage into the cell, enzymatic inactivation of drugs, or even modification of cellular structure interfering in the drug-bacteria interaction. There are still plenty of new genes out there in the environment that can be appropriated by putative pathogenic bacteria to resist antimicrobial agents. On the other hand, there are several natural compounds with antibiotic activity that may be used to oppose them. Antimicrobial peptides (AMPs) are molecules which are wide-spread in all forms of life, from multi-cellular organisms to bacterial cells used to interfere with microbial growth. Several AMPs have been shown to be effective against multi-drug resistant bacteria and have low propensity to resistance development, probably due to their unique mode of action, different from well-known antimicrobial drugs. These substances may interact in different ways with bacterial cell membrane, protein synthesis, protein modulation, and protein folding. The analysis of bacterial transcriptome may contribute to the understanding of microbial strategies under different environmental stresses and allows the understanding of their interaction with novel AMPs.

## Introduction

According to our recent history, human activity has markedly enhanced the evolution and distribution of resistant bacteria worldwide both in hospitals, human, and animal communities, and in the open environment, although this human activity is not necessarily the only, or even the proximate, cause for antimicrobial resistance phenomenon (Josephson, 2006; Wright, 2010). In this regard, most of the scientific research in antibiotic resistance over the past six to seven decades has been focused on association of drug-resistance with pathogenic bacteria. Given what we now know about the dispersal of resistance genes in nonpathogenic bacteria, this focus on pathogens actually neglects the majority of genes associated with resistance (D'Costa et al., 2006).

Since its use as a therapeutic tool to fight infectious diseases was proposed, antimicrobial drugs have reduced the mortality, but not the persistency of infectious diseases. Due to their use and misuse, these drugs have stimulated bacterial evolution toward the development of resistance, as an adaptive mechanism to the environment. While the selective pressure is maintained, adaptive mechanisms are transmitted to new generations, through the genetic flow. The phenomenon has acquired considerable importance in public health (Levy, 1998). The resistance may be associated with chromosomal mutations or imported genes through genetic recombination. In antimicrobial resistant microorganisms, resistance genes such as plasmids, transposons, and integrons can be inserted into the chromosome or extrachromosomal genome. Resistance may also be associated with a general impermeability of the bacterial cell envelope (El-Halfawy and Valvano, 2012).

The development of microbial resistance to antimicrobials had been going on in nature long before antibiotics were made available to chemotherapy. It is recognized that bacteria, including human pathogens, may acquire resistance genes in natural environments, particularly in soils (Josephson, 2006; Wright, 2007). Taking the recent methodological approaches, the concept of the antibiotic resistome has been advanced to serve as a framework for understanding the ecology of resistance on a global scale (Wright, 2007).

The resistome consists of a collection of all antibiotic resistance genes including those circulating in pathogenic bacteria, antibiotic producers, and benign non-pathogenic organisms found either free living in the environment or as commensals of other organisms (D'Costa et al., 2006). Most of the so called antibiotic producers live in soils, and as an ecological consequence, most of the susceptible bacteria in their vicinity, including human pathogens, die off, but some develop resistance to these natural products thought of control the microbial population (Wright, 2010; Cox and Wright, 2013).

The limited number of antibacterial classes and the common occurrence of cross-resistance within and between classes have also reinforced the urgent need to discover new compounds targeting novel cellular functions not yet targeted by currently used drugs (Chung et al., 2013). Bacteria are known to employ different strategies for antibiotic resistance. Resistance may be acquired by spontaneous mutation in the coding gene of the target protein resulting in no or reduced affinity to the antibiotic or by horizontal transfer of antibiotic resistance genes from other bacteria (Hassan et al., 2012).

An antibiotic-resistance gene product may act by enzymatic degradation of the antibiotic, by altering the antibiotic target site or by pumping the incoming antibiotic out of the cell by a transport mechanism. Such processes make infection treatment very difficult as we face sophisticated, highly resistant and often multi-resistant pathogens such as *Pseudomonas aeruginosa* (Paterson, 2006), *Escherichia coli* (Overbye and Barrett, 2005), methicillin-resistant *Staphylococcus aureus* (MRSA) (Reynolds et al., 2004) and penicillin-resistant *Streptococcus pneumonia* (Karchmer, 2004).

The antimicrobial peptides (AMPs) is a class of molecules that may be used to overcome the bacterial resistance challenge. Their occurrence is a wide-spread phenomenon in all forms of life, from multi-cellular organisms to bacterial cells. In higher organisms, AMPs contribute to innate immunity and are part of the first defense line against harmful micro-organisms. In bacteria, production of AMPs provides a competitive advantage for the producer in certain ecological niches because the peptide mediates the killing of other bacteria (Hassan et al., 2012). They are constitutively expressed or induced by endogenous or exogenous elicitors, such as developmental stage or pathogen predation (Sachetto-Martins et al., 2000). AMPs are small proteins 20–50 amino acid residues long, often having common properties such as the small number of amino acid residues, cationicity, and amphipathicity (Tavares et al., 2008). The AMPs interact with membranes in different ways, but in general three different models have been used to define their mode of actions in model membrane systems. In the barrel-stave mechanism, peptides integrate into the membrane and form membrane-spanning pores. In the toroidal-pore mechanism, AMPs form membrane-spanning pores together with intercalated lipids. And in the carpet mechanism, peptides accumulate on the membrane surface in a carpet-like manner and at a threshold density so that they dissolve the membrane without forming transmembrane channels (Pietiäinen et al., 2009; Brogden, 2011). However, membrane damage is not the single mechanism whereby AMPs cause cell death. They may also affect functions of several other cell components and act as metabolic inhibitors of cellular processes including biosynthesis of the cell wall, nucleic-acids and proteins. In these cases, the cell death can be the result of multiple inhibitory effects (Brogden, 2005).

AMPs show broad-spectrum antimicrobial activities against various microorganisms, including Gram-positive and Gram-negative bacteria, fungi, and viruses. Many AMPs are effective against multi-drug resistant (MDR) bacteria and possess low propensity for developing resistance probably due to their distinguished mode of action (Seo et al., 2012). AMPs could be very diverse in sequence and structure but most of them are positively charged, allowing their interaction with the bacterial envelope. These peptides are active at very low concentrations (micromolar to nanomolar range) and most of them kill their target microorganism via a non-receptor mediated mechanism involving permeation of the target membrane (Guralp et al., 2013).

AMPs can be classified into four groups based on their structures: α-helical peptides, β-sheet peptides, extended peptides, and loop peptides (Nguyen et al., 2011; Fjell et al., 2012). Understanding the structure-activity relationships (SAR) of AMPs is essential for the design and development of novel antimicrobial agents with improved properties. In particular, the atomic level structures of AMPs can provide versatile information for all stages of drug development, including the peptide design and modification for pharmaceutical application (Seo et al., 2012).

Microbial pathogens have evolved different systems to resist the effect of antimicrobial peptides. These mechanisms can involve the destruction of antimicrobial peptides (by proteolytic digestion), change of antimicrobial peptide target (i.e., the microbial membrane), and removal of antimicrobial peptides from their site of action (through efflux pumps or by alteration of the cell surface composition) (Rio-Alvarez et al., 2012). The modifications of lipopolysaccharide (LPS) to mask the negative charges that allow interaction with AMP are one of the main responses to these compounds in many Gram-negative bacteria (Costechareyre et al., 2013).

Some bacteria such as *Staphylococcus enterica* serovar typhimurium exhibit a regulatory system controls virulence that is involved in the regulation of Mg^2+^ uptake systems, survival in macrophages and resistance to antimicrobial peptides (AMP). Several enzymes, encoded by *pagP, pagO, pmrC, pmrG, lpxO, pmrHFIJKLM*, modify LPS, mostly by adding or modifying palmitate, phosphoethanolamine or 4-aminoarabinose to mask negative charges that allow interaction with cationic AMPs (Costechareyre et al., 2013). Costechareyre et al. (2013) using *Dickeya dadantii*, which is an insect and plant pathogen, to understand the regulation of genes involved in response to AMPs, observed that through transcriptome different genes are involved in response to AMPs when the bacteria infect the aphid (*Acyrthosiphon pisum)* and plant.

Antimicrobial peptides (AMPs), particularly the so-called bacteriocins produced by bacteria, may be an important contributor in this context as they often have a relatively narrow killing spectrum which comprises mostly bacteria closely related to the producers (Hassan et al., 2012).

Many hundreds of different peptides, differing in size, charge, hydrophobicity, conformation, primary structure, as well as in post-translational modifications, have been demonstrated in frog defensive skin secretions (Evaristo et al., 2013).

The knowledge about AMP action mode and resistance mechanisms shared by different microorganisms may point the direction for discovery and design of new drugs.

## New approaches to AMPs resistance

The knowledge acquired in the last two decades concerning the evolution of antimicrobial resistance to widely prescribed drugs, and the search for new antimicrobial candidates such as AMPs, thought to be natural barriers against bacteria, eukaryotic parasites, viruses, and fungi, has resulted in a better understanding of how microorganisms have become resistant to these proteins (Marshall and Arenas, 2003; Wilcox, 2004; Hancock and Sahl, 2006; Perron et al., 2006).

The variety of already described antimicrobial peptides related to the different sequences, shows that the same peptide sequence is rarely associated with two different species, even closely related. Several multicellular organisms express a collection of peptides of different chemical structures, as a local defensin (Zasloff, 2002). However, despite the structural diversity, most of the already sequenced antimicrobial peptides show at least 50% hydrophobic amino acid residues and a low proportion of both neutral polar and negatively charged amino acids (Hancock and Chapple, 1999). It is accepted that this structural skeleton may explain why the majority of AMPs persists at water-lipid interfaces and then disturb microbial membrane components (Ruissen et al., 2001). Membrane damage is considered the primary antimicrobial mechanism of the so called cationic antimicrobial peptides (CAMPs) or ribosomally synthesized antimicrobial peptides (RAMPs) (Perron et al., 2006), and requires interaction with microbial membrane lipids and hydrophobic properties to enable integration of the peptide into the hydrophobic core of the membrane (Peschel and Sahl, 2006).

Studies with CAMPs thrombocidins, defensins, and cathelicidins show a potential use as skin and epithelia protectors against invading microorganisms, such as *Staphylococcus aureus* and *Salmonella enterica*, by reducing the net negative charge of the bacterial cell envelope through covalent modification of anionic molecules (e.g., teichoic acids, phospholipids, and lipid A) resulting in repulsion of CAMPs. Other mechanisms have also been reported such as expelling CAMPs through energy-dependent pumps, altering membrane fluidity and CAMPs cleavage with proteases (Peschel, 2002; Marshall and Arenas, 2003).

Although nonspecific targets led researchers to suggest that it would be difficult for the bacteria to develop resistance to some peptides (Ge et al., 1999a,b; Schroder, 1999; Zasloff, 2002; Boman, 2003; Jenssen et al., 2006), molecular mechanisms of resistance to CAMPs have been suggested in several groups (Zasloff, 2002). In *S. aureus*, changes in the cell wall appear to involve the operon *dltABCD*, which results in carriage of positively charged D-alanine from the cytoplasm to anionic teichoic acids (Peschel et al., 1999; Kristian et al., 2003; Nizet, 2006). Perron et al. (2006) have studied the effects of resistance to pexiganan, CAMP analog of magainin, in different bacterial strains (mutants for *mutS* and *mutL* genes—*Pseudomonas fluorescens* and *Escherichia coli*) and observed MIC50 increased in both mutant strains. They also observed a reduction in the lag phase after subsequent growth in pexiganan presence. The contribution of these resistance mechanisms in bacterial pathogenesis may be confirmed by studies with mutants. It is accepted that such prospective investigations are of extreme relevance, since these potential AMPs are thought to be an alternative to well established antibiotics used in chemotherapy against multiresistant bacteria (Nizet, 2006; Brogden and Brodgen, 2011; Maróti et al., 2011). Mechanisms such as peptidases production, down regulation of host AMP production, and cellular filamentation have also been related (Nizet, 2006; Maróti et al., 2011).

AMPs may interact with intracellular targets, binding to DNA, RNA and protein, or even interfering with the characterized *FtsZ* gene, responsible for bacterial cell division septum or with protein synthesis such as DNA gyrase and DnaK (Brogden, 2005; Chauhan et al., 2006; Handler et al., 2008; Maróti et al., 2011). Genetic markers related to the defensins and cathelicidin mediated AMPs resistance include *kasB* in *Mycobacterium marinum* (Gao et al., 2003), *sak* in *S. aureus* (Jin et al., 2004)—for defensins; and *emm1* in Group A *Streptococcus* (Lauth et al., 2009).

Additionally, some AMPs have non-protein targets such as the peptidoglycan precursor lipid II and ATP (Hilpert et al., 2010; Sass et al., 2010). Modifications on cell surface have also been correlated with the AMPs resistance and several genetic markers have already been described, such as *mprF/lysS* in *S. aureus* (Peschel et al., 2001; Nishi et al., 2004), *dlt* operon in Group B *Streptococcus* and *Listeria monocytogenes* (Abachin et al., 2002; Poyart et al., 2003), *htrP* in *Haemophilus influenzae* (Starner et al., 2002), *pmr* in *Pseudomonas aeruginosa* (Moskowitz et al., 2004).

The active efflux of AMPs has already been observed and might be related to different genetic markers in various bacteria species, such as *mtr* in *Neisseria gonorrhoeae* (Jerse et al., 2003), *sap/sapA* operon in *S. enterica* and *H. influenzae* (Parra-Lopez et al., 1994; Mason et al., 2005) and *qacA* in *S. aureus* (Kupferwasser et al., 1999).

Moreover, the degradation of AMPs has being correlated to several genetic markers: *lasB* in *P. aeruginosa* (Schmidtchen et al., 2002), *gelE* in *Enterococcus faecalis* (Schmidtchen et al., 2002), *zapA* in *Proteus mirabilis* (Schmidtchen et al., 2002), *speB*/*ideS* in Group A *Streptococcus* (Schmidtchen et al., 2002), *aur* gene in *S. aureus* (Sieprawska-Lupa et al., 2004), *degP* in *Escherichia coli* (Ulvatne et al., 2002), and *rgpA/B* in *Porphyromonas gingivalis* (Devine et al., 1999).

The use of AMPs as pharmaceuticals will promote selective pressure for bacterial strains that are resistant also to the repertoire of host-defense peptides in the human body (Bell and Gouyon, 2003; Nizet, 2006). In this context, the bacterial resistome must also consider endogenous housekeeping genes which may interact with the AMPs. To select genetic markers related to the bacterial resistome in this holistic point of view remains as an important challenge (Islam et al., 2001; Taggart et al., 2003; Wright, 2007). To illustrate the role of the housekeeping genes in the AMPs resistance, several authors have reported the importance of regulatory genes such as *phoP/phoQ* in *S. enterica* and *P. aeruginosa, pmrB* in *P. aeruginosa*, and *rpoE* in *S. enterica* (MacFarlane et al., 2000; Ernst et al., 2001; McPhee et al., 2003; Crouch et al., 2005).

Considering different organisms, such as fungi, a lot is known about the mechanism of resistance to antimicrobial drugs, but there are few reports on AMPs resistance. So far, AMPs in these organisms include modification of *erg11/mdr1* gene and *pdr5* locus, over expression of specific drug efflux pumps, alteration in sterol biosynthesis and alteration in AMP target, AMP inactivation and reduction in the intracellular concentration of target enzymes (Ghannoum and Rice, 1999; Balkis et al., 2002; Gulshan and Moye-Rowley, 2007). As observed for bacteria, antifungal drug resistance is quickly becoming a major problem, especially considering the expanding population of immunocompromised patients who have contributed to an increased incidence of opportunistic and systemic fungal infections.

With regards to the antifungal drug resistance mechanisms, the genetic markers codifying for multidrug efflux pumps and their upregulation have been highlighted (Balkis et al., 2002; Gulshan and Moye-Rowley, 2007). Jabra-Rizk et al. (2004) described two different types of efflux pumps in *C. albicans* and *C. dubliniensis*: adenosine triphosphate–binding cassette (ABC) transporters encoded by the *cdr* genes (*CDR1* and *CDR2*) and major facilitators encoded by the *mdr* genes.

Overall, it is accepted that further prospective studies on antimicrobial resistance are needed to enable a better understanding of the microbial genetic diversity that underlies resistance. Such knowledge will help and guide our efforts to develop new potential drugs to overcome the resistance phenomenon (Wright, 2007).

## Next generation sequencing and AMP prediction

**Figure 1:**
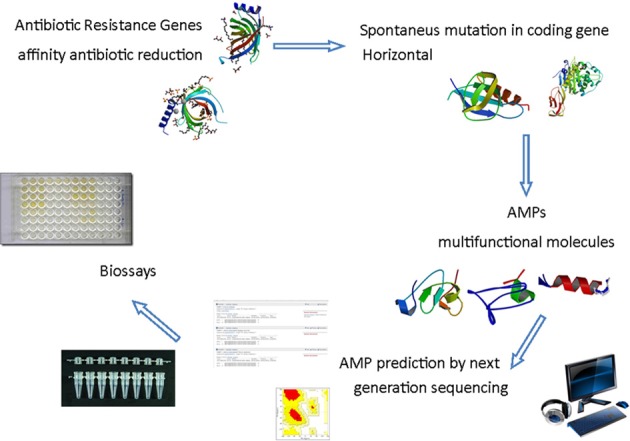
**Schematic antimicrobial peptides prospecting from the *in silico* analysis of sequences obtained by next generation sequencing.** The demand for new antimicrobials to prevent microbial resistance from multifunctional action encourages the search for AMPs through prediction for next-generation sequencing followed by analysis of bioinformatics and computational modeling in order to produce effective peptides after antimicrobial trials. PDB IDs 3GP6; 1THQ; 2JSO; 3HUM; 2L24; 2KUS; 2LB7; 2NY8.

The next generation sequencing technologies have opened the opportunity to access genomes and transcriptomes at high throughput level allowing the researchers to understand a wide variety of physiological response of various types of organism. As a consequence new tools are available for antimicrobial discovery and design (Figure 1). The knowledge of host resistance mechanisms vs. susceptibility is important to the development of new approaches to prevent and/or treat human infectious diseases (Teles et al., 2013). The innate immune response in different organisms has the potential to reveal new and/or novel molecules for antimicrobial purpose. During transcriptome analysis of the oral chicken *Salmonella* infection four steps were observed and none of the genes was directly involved in bacterial infection, but associated with inflammatory response (Matulova et al., 2013). On the other hand persistence of *Salmonella* in several other niches is observed by resistance to AMPs and its sensibility is increased by adrenaline, down regulating the promoter of the *pmr operon* that controls resistance genes to AMPs (Karavolos et al., 2008). The combination of transcriptome and proteomic strategies were used to study the Australian scorpion, revealing that the molecular weight found for proteomics analysis was not completely adjusted to amino acid sequence deduced from cDNA cloned genes. Some reasons are pointed out by the authors: the level of gene expression is not necessarily the same information obtained from the cDNA, posttranslational modifications, or sample preparation (Luna-Ramírez et al., 2013). Regardless of problems encountered, some potential therapeutically peptides were identified in those samples. In ladybird *Harmonia axyridis* the successful invasive behavior was revealed by 454 sequencing. The two layer innate immune system is composed of a chemical weapon, mediated by the secondary metabolite harmonine, associated with a wide range of AMPs resulting from multiple gene duplication and divergence events (Vilcinskas et al., 2013). In the scorpion *Hetermetrus petersii* its venon showed four families of antimicrobial and cytolyc peptides identified by 454 sequencing platform (Ma et al., 2010). In *Spodoptera exigua* larvae upon AcMNPV infection the 454 analysis demonstrated that some genes, including genes encoding for AMPs, are down regulated (Choi et al., 2012). Summarizing, the association of transcriptome and proteomics technologies offers new points of view for AMP mode of action in different organisms, showing different potential and different strategies for prospection. In the bivalve mollusk *Ruditapes philippinarum*, for example the use of 454 platforms allowed the identification of 36 AMP sequences (Moreira et al., 2012). The analysis of transcriptome of the American dog tick infected with different microorganisms allowed the researcher to identify a novel elicited defensin in the Arachnids immune system response transcripts (Jaworski et al., 2010). Facing up to the high diversity of organisms, various tissues and physiological approaches, the number of novel and new AMPs derived from biodiversity is a vast field for research.

## Prediction of antimicrobial peptide from DNA/RNA library

### Antimicrobial peptides search tools

The antimicrobial peptides are directly related to the innate and acquired immune response of organisms, and their potential to kill microorganisms resistant to many antibiotics has attracted the interest of the pharmaceutical industry. In this aspect tools to find and produce antimicrobial peptides have created a revolution in research for new drugs. According to Belarmino et al. (2010), the development of bioinformatics tools for predicting patterns in biological sequences has already allowed a routine search in databases of ESTs (Expressed Sequence Tags) of plants by defensins and a subsequent validation by antimicrobial testing.

The APD2 (Antimicrobial Peptide Database Second Version) is one of the main databases of antimicrobial peptides, allowing users to search for families of peptides, post translational modified peptides, among other options (Wang et al., 2009). APD provides an option to calculate and to predict AMPs in order to extract important information about peptides such as total charge, hydrophobic rate, in addition to providing an alignment with deposited sequences (http://aps.unmc.edu/AP/main.php). The information provided can be linked to the data on the hydrophobic moment calculated by the web-program HydroMcalc (Tossi et al., 2002) available at http://www.bbcm.univ.trieste.it/~tossi/HydroCalc/HydroMCalc.html, allowing the prediction of antimicrobial peptides.

In addition to the APD, another database of AMPs is the CAMP (Collection of Antimicrobial Peptides) available at http://www.bicnirrh.res.in/antimicrobial. Such tools provide information related to the sequence, definition of protein biological activity, taxonomy of the source organism, target organisms—indicating the MIC (minimum inhibitory concentration), hemolytic activity of the peptide and links to external databases such as SwissProt, PDB, PubMed and the NCBI Taxonomy (Thomas et al., 2010). The iAMP-2L, available at http://www.jci-bioinfo.cn/iAMP-2L, is a web-server used for the prediction of uncharacterized sequences as antimicrobial. Once the subject sequence is identified as an antimicrobial, the server indicates to which class (antibacterial, anticancer, antifungal, anti-HIV and anti-viral) the peptide belongs. Peptides are promiscuous molecules (Franco, 2011) and are invariably classified in more than one class and family (Xiao et al., 2013). All of these characteristics reinforce how these new technologies can make available an unlimited source for new drugs and biologically active molecules. Tools of bioinformatics for modeling are fundamental for development of this research area.

### Types of modeling and predicting structures applied to antimicrobial peptide

In the following steps we present a summary of a method to develop such peptides that can predict the structure of peptides and proteins in two ways: experimentally, through methods such as nuclear magnetic resonance (NMR), X-ray diffraction and crystallography, and theoretically, by computational modeling methods, which involve *comparative modeling*, *threading (folding) modeling* and *ab initio* (*de novo*). Experimental methods of peptide prediction and modeling have typical difficulties. The lack of structural conformation of plant bactericidal peptides prevents more detailed classification of AMPs (Porto and Franco, 2013). The use of computational tools and methods has become an important strategy in the search for bioactive peptides. However, there are still some limitations in this prediction method, such as the difficulty of developing a general method for predicting the nature and activity of antimicrobial peptides, due to low homology sequences that can occur (Lata et al., 2007; Torrent et al., 2012).

#### Comparative modeling

Comparative homology modeling is a method based on the structures similarity, i.e., similar amino acid sequences tend to have a very similar secondary structure. Thus, it is possible to use as a template structures solved by experimental means, in order to predict the 3D conformation of peptides and proteins using computational algorithms. In comparative modeling, the alignment of the sequence to be predicted and the template must present an identity of at least 30% (Baker and Sali, 2001), a large number of cases with alignments with low identity between target and template can lead to better models of 3D structure (Rayan, 2009). In fact the identity of the alignment can be put aside when we are in a situation of functionality. For example, imagine a protein A having 90% identity to another protein B, but a different function, and also a protein C that has 70% identity and the same function as B. In this case protein C would be the best template and B is not the shape desired. The alignment score should also be considered when working with whole proteins, since in certain programs, such as BLAST, alignments may appear above 30%, but still low coverage.

The *in silico* prediction method by homology modeling is divided into four main steps: (1) identification of structures and selection of templates, (2) alignment of the target sequence with the chosen model structure, (3) generation of models for the target structure, using information about the structure of the template; (4) validation of the models generated for the target (Martí-Renom et al., 2000). It can also be interesting to implement a fifth step which is the model of refinement by energy minimization, which is important in the context that during the production of the geometric errors can occur in regions of the main chain (Vyas et al., 2012).

The first two steps to create and predict a three-dimensional model of a protein or peptide involve query of database structures experimentally determined by crystallography techniques, X-ray diffraction or nuclear magnetic resonance (Kiefer, 2012). First, research tools in the databases such as BLAST (Basic Local Alignments Search Tool) that allow local alignment. Searching sequence of regions similar to other regions of sequence is an essential step to find sequence templates. The BLAST minimizes the time spent on research, discarding alignments in which regions between the query and subject sequences have few chances to exceed a pre-determined score (Altschul et al., 1990). During the search for similar sequences in databases, attention should be given to the best method, i.e., one that is both sensitive (able to identify sequences related bit) and selective (relations between the query and subject sequences are true). At this point it is worth mentioning one of the most used tools in the search for similar sequences, PSI-BLAST (Position-Specific Iterated Basic Local Alignment Tool) from NCBI, which differs from conventional BLAST due to its higher accuracy and greater statistical sensitivity (Li et al., 2011).

After choosing sequences of templates that will be used in the second stage, the optimal alignment between this sequence and the target is required to build three-dimensional models (Centeno et al., 2005). The main strategies used are: progressive alignment between the sequences using the software Clustal W (Larkin et al., 2007), the sequence-profile alignment, HMM-based method (HIDDEN MARKOV MODEL) between query and profile of families of templates, using up the database profiles Pfam (Finn et al., 2010) and HMMER web server (Finn et al., 2011) one can still perform profile-profile alignment, from building a profile for the target and matching with the profile templates in a database of profiles (Centeno et al., 2005; Ramachandran and Dokholyan, 2012; Venclovas, 2012).

The models are generated based on the structural information provided by the template and the sequence alignment between them and predicted (Kiefer, 2012). Currently there are several programs and web servers that can be used to build models of proteins and peptides, the main one is Modeler, developed by Sali and Blundell (1993). The Modeler is used to compare the target structure by satisfaction of spatial constraints involving restrictions on atomic distances, angles dihedral, and stereochemistry. The information modeler generated is also combined with the statistical calculation preferences of constraints derived from the sequence template homology (Eswar et al., 2003; Vyas et al., 2012). Another tool also used in model building by homology is SwissModel, which unlike the modeler searches in a homologous target database with the BLAST protein and then determines a three dimensional model, finding the core backbone and modeling loops and chain laterals (Schwede et al., 2003).

In homology modeling each step is directly linked to the previous, so in the event of accidental errors, these can be propagated. Thus, it becomes necessary to validate the final model and interpretation of the target. The generated model can be validated as a whole or for individual regions (Martí-Renom et al., 2000), the basic need is for a good model built on good stereochemistry (Hillisch et al., 2004). The main tools for analysis and validation of models generated by comparative modeling are: Procheck (Laskowski, 1993) and Molprobity (Chen et al., 2009), both for quality analysis stereochemistry; Whatchek (Hooft et al., 1996) and Qmean (Benkert et al., 2009) used to evaluate the quality of the model, and the ProsaWeb (Wiederstein and Sippl, 2007) used in the analysis of interaction energy between the model residuals. According to Martí-Renom et al. (2000), the most common errors that may occur during modeling are positioning errors of side chain distortions in regions aligned, regions with an inefficient mold alignment and wrong choice of template.

The refinement of the model in general uses methods of molecular dynamics calculations of force fields, the most common being the CHARMM (Brooks et al., 1983) and GROMOS (Schuler et al., 2001). A refinement process can be defined as walking on the surface of covalent and hydrogen bonds in the model, the search for a better minimum energy than the energy of departure, therefore, a difficult task (Gront et al., 2012). The energy minimization can promote excessive deviation of the model structure, compared to the original, which actually is not ideal; therefore, you should keep the number of cycles of minimization to a minimum, which is sufficient for improved stereochemistry of the model (Peitsch, 2002).

#### b-Threading modeling

The modeling threading or by folding pattern recognition is a method of predicting three dimensional structures by looking for folding patterns, applying the combination-linear alignments profile and adjusting the profile structure of the target reference frames (obtained from folding profile libraries).

The LOMENTS is a meta-server which includes nine major servers threading (PPA-I, SP3, PPA-II, sparks2, PROSPECT2, FUGUE, HHSEARCH, PAINT, SAM-T02), allowing the selection of models through research for 30 models for each of the individual servers, excluding short alignments, and defining models of greater structural similarity (Wu and Zhang, 2007). It is an important tool for the study of structures in modeling template folding. Among commonly used tools in modeling protein folding are the ROSETTA (Kaufmann et al., 2010) and I-TASSER (Wu et al., 2007; Roy et al., 2010). Such tools have their operation based on either amino acid sequence of the target and information about structures in the template experimentally resolved, or using predictors of secondary structure and folding as mentioned above, with libraries of fragments.

#### c-Ab initio modeling

This method of predicting three-dimensional structures ignores in principle the use of reference structures solved experimentally. Prediction *ab initio* (*de novo*) makes use of the energy minimized functions and research of spatial conformations that the target can take, and this is important for the use of force fields and methods of molecular dynamics and Monte Carlo simulations (Lee et al., 2009). According to Helles (2008), the three factors that make *ab initio* interesting for homology modeling, are that this does not provide accurate information about how a given protein or peptide acquires structure, many proteins and peptides do not have sufficient (> 30%) experimentally solved homology molecules, and even if the target presents high similarity with templates, it does not mean they will present the same structural profile.

*ab initio* Software such as ROSETTA and I-Tasser, cited above, have been used as *de novo* prediction programs (Wu et al., 2007; Kaufmann et al., 2010). However, by considering information frames of reference they are not actually *ab initio* techniques. A tool that is completely is LINUS (Local Independently Nucleated Units of Structure), which does not make use of structures or reference sequences, initiating the construction of the target from the extended chain as a result. The simulation performed by the software promotes the disruption of conformations of three randomly chosen residues, and evaluates the energy, using Monte Carlo procedure to validate the favorable conformation predicted (Srinivasan and Rose, 2002). Another tool also used currently is the QUARK (http://zhanglab.ccmb.med.umich.edu/QUARK/), a tool that builds models from small fragments (residues 1–20) using Monte Carlo simulations (Xu and Zhang, 2012).

The intensive growth of research of AMPs and development of robust databases the discovering of novel and new biological active peptides (Amaral et al., 2012). The development of antimicrobial peptides from genomic and transcriptome databases can be an alternative strategy to the studies with research and development of AMPs.

## Modification medicines

As long as antimicrobials were made available in the 1940s, there were no concerns related to the antimicrobial resistance mechanisms. However, the discovery of other antimicrobial agents and even the modification of those already described were not able to stop microbial evolution, such as the rapid emergence of β-lactamase-producing *Staphylococcus aureus* strains (Spellberg, 2009; Theuretzbacher, 2009; Choffnes et al., 2010).

The accelerated increase and global expansion of bacterial resistance made it necessary the search for new fighting agents (Spellberg, 2009; Choffnes et al., 2010). One of the main factors associated with this increasing antimicrobial resistance was the misuse of antimicrobials (Gwynn et al., 2010).

Driven by high profitability, the pharmaceutical industry has focused its production on blockbuster drugs (or global FMCG)–such as those used in the treatment of chronic diseases such as cancer or sexual dysfunction, for example—rather than the development of antimicrobial drugs used for short term treatment of acute infectious diseases (Theuretzbacher, 2009).

The economic advantages offered by blockbuster drugs coupled with the high cost of production and the low economical income related to the antimicrobial production, if compared to the profitability of other drug production, led to a lack of investment in the development of new antimicrobial agents in the 1990s (Spellberg, 2009; Theuretzbacher, 2009). In this regard, the production of new antibiotics becomes, now, very expensive due to the rationale and steps of manufacturing and preclinical testing and clinical trials, up to their insertion in the market. The searches for new agents has to overcome the mechanisms of bacterial resistance, and therefore, are based on the search for new routes of administration, new targets or mechanisms of action toward the same target, which ends up limiting the production of effective potential agents (Gwynn et al., 2010). Add to that the availability of generic formulations and the development of drugs kept to treat only severe diseases to avoid quick bacteria resistance development, further also contributed to the economical failure related to new antimicrobial releases (Spellberg, 2009). In this point of view the *in silico* prediction of antimicrobial peptides becomes an advantage for industry due to low cost and time consumption.

Furthermore, the wide use of broad-spectrum antimicrobials has contributed to the need for new drugs given the emergence of the so called multi-drug resistant bacteria (MDR) (Choffnes et al., 2010; Gwynn et al., 2010). The decline in production of new agents was compounded by the loss of effectiveness of existing antimicrobials without a concomitant replacement by new therapeutic options. In a study by Shlaes and Moellering (2002), the medical community was alerted to the lack in new drug discovery, and the authors concluded that the development of new antibacterial agents was even lower than that related to hyperactivity disorder and male erectile dysfunction (Spellberg, 2009).

Within the current scenario of increasing bacterial resistance, however, it becomes necessary to resume production of new antimicrobial agents, or discussion of new strategies for the use of the available drugs. This discussion has motivated and encouraged scientific research on the subject, in order to decrease the cost of production within large pharmaceutical companies (Spellberg, 2009). For example, while other drugs require 15 candidates to yield one FDA-approved product, antibiotics require 72 candidates to yield an FDA-approved product, which currently costs 400–$ 800 million per approved agent (Spellberg et al., 2008; Forsyth, 2013; IDSA, 2013). The production of antimicrobials is not profitable also because: the drugs are used for a short period of time (7–14 days), sold for low price and prescription controlled market (Forsyth, 2013). As a result, it is estimated that about two million Americans per year develop hospital infections, mostly caused by multidrug-resistant bacteria pathogens, which increases treatment costs in about U$ 21 million to U$ 34 billion, compared to antibiotic-susceptible pathogens (Roberts et al., 2009; Spellberg et al., 2011). Nosocomial infections such as pneumonia and sepsis, killed about 50,000 Americans in 2006 and cost to the US health care system more than U$ 8 billion (Eber et al., 2010).

To encourage the production of new antimicrobials, Government policies have been issued, such as the GAIN (Generating Antibiotics Incentives Now) Act, which states: (i) warranty for new approved drugs protection from competition in the marketplace by limiting FDA approval of similar drugs during the a certain exclusivity period; (ii) review and fast-rack approval priority for qualified antimicrobial drugs, antibiotic applications will be eligible for both priority review and fast-track approval through the FDA new drug application process; and (iii) study of incentives for Qualified Infectious Diseases Biological Products, to encourage research, development, and marketing for qualified infectious disease biological products (Forsyth, 2013). Besides this, proposals have been discussed for new ways of using drugs already known and established for the microorganisms which have been made resistant (Spellberg, 2009).

In this regard, considering the evolution of bacterial pathogens associated with infectious diseases today, the need to develop new agents to control multiresistant bacteria is presumed, or to prospect new ways of using the inefficient well-established antimicrobial arsenal, aiming to overlap the existing limitation in antibacterial chemotherapy (Rai et al., 2009; Spellberg, 2009; Choffnes et al., 2010).

With regards to the AMPs and their eventual modifications as an alternative strategy to overcome the need of new drugs, it is important to undergo a retrospective analysis of the co-evolution of antimicrobial peptides and bacterial resistance. Initially several peptides had been reported in the scientific literature and among them, cationic peptides called attention by their mechanisms of action: using positively charged molecules, amphiphilic, with affinity to bacterial membranes. However, during initial *in vitro* and in preclinical trials, resistant strains have been noticed. Overall, variations in the peptide sequences are proposed leading to conjugate molecules (Peschel and Sahl, 2006).

Obtaining AMPs can be performed in three different ways: direct isolation of the producer, by chemical synthesis or recombinant expression (Li et al., 2010; Parachin et al., 2012). Modifications in AMP composition, structure and function are being used to create more stable molecules. Six distinctive new classes of AMPs have already been reported (Brogden, 2011; Brogden and Brodgen, 2011; Tossi, 2011). The first class includes mimetic peptides, which are non-peptidic, synthetic molecules, which mimic the natural properties of AMPs. Its structure requires a different composition such as peptoids, arylamides oligomers, β-peptides, or phenylene ethynylenes (Rotem and Mor, 2009). The second class includes hybrid peptides, AMPs constructed of the active regions of two to three peptides, such as cecropinA-melittin (CEME/ CEMA/ CP26/ CP29) (Piers and Hancock, 1994). The potential benefits of each individual fragment are combined to increase antimicrobial activity, reduce antimicrobial spectrum of activity or reduce cytotoxicity for host cells. The third class includes peptide congeners, a chemical compound closely related to another in composition, such as congeners of CAP18, LL-37, SMAP28, ovispirin, and Q25. They may contain changes in tertiary structure, change of specific amino acids in the sequence to load change, among other characteristics. The fourth class includes cyclotides and stabilized AMPs. Cylotides are cyclopeptides with a head-to-tail cyclic backbone, containing 30 amino acid residues with three conserved disulfide bonds (i. e., cyclized angiotensin and cyclic diastereomeric lysine ring) (Ireland et al., 2010). The fifth class includes peptide conjugates which is connected to micelles, liposomes, antibodies, steroids or fatty acids, such as lactoferrin—lauric acid (Chu-Kung et al., 2010), and the sixth class includes immobilized peptides via incorporation into distinct materials or absorbed to a variety of surfaces where they still retain their ability to bind and kill bacteria. These groups of new peptides have a variety of potential medical and industrial applications in many different areas (medicine, veterinary, agriculture, pharmaceutical, food) (Costa et al., 2011).

In conclusion, the misuse of anticrobials lasting recent decades has increased the spread of mutations allowing the development of multidrug resistant microbes. The antimicrobials were neglected due to economic interest. Thus, for infectious diseases the development of new antimicrobial with low cost and broad spectrum of action becomes of great importance, because the lifetime of such molecules is very short and a wide range of molecules is important to overcome the novel resistant pathogens. The molecular modeling of AMPs from transcriptome has arisen in current times as an important alternative for drug development.

## Financial support

Fundação de Amparo à Pesquisa de Minas Gerais (FAPEMIG).

### Conflict of interest statement

The authors declare that the research was conducted in the absence of any commercial or financial relationships that could be construed as a potential conflict of interest.
